# Intra-abdominal *EWSR1/FUS-CREM*-rearranged malignant epithelioid neoplasms: two cases of an emerging aggressive entity with emphasis on misleading immunophenotype

**DOI:** 10.1007/s00428-021-03140-3

**Published:** 2021-07-06

**Authors:** Abbas Agaimy, Robert Stoehr, Mike Otto, Jan Hinrich Bräsen, Nicole Pfarr, Björn Konukiewitz, Atsuko Kasajima, Arndt Hartmann, Günter Klöppel

**Affiliations:** 1 Institute of Pathology, Friedrich-Alexander University Erlangen-Nürnberg (FAU), University Hospital Erlangen (UKER), Erlangen, Germany; 2 Comprehensive Cancer Center Erlangen-EMN (CCC ER-EMN), Erlangen, Germany; 3 Medical Center of Histology, Cytology and Molecular Diagnostics Trier LLC (GmbH), Trier, Germany; 4 Institute of Pathology, Hannover Medical School, Hannover, Germany; 5 Institute of Pathology, Technical University Munich, Munich, Germany

**Keywords:** RCC, ALK, EWSR1, CREB, CREM, FUS, Primary renal sarcoma, Mesentery, Neuroendocrine, Paraganglioma

## Abstract

CREB family (*CREB1*, *ATF1*, and *CREM*) gene fusions are defining markers in diverse mesenchymal neoplasms (clear cell sarcoma, angiomatoid fibrous histiocytoma, and others). However, neoplasms harboring *EWSR1-CREM/FUS-CREM* fusions are rare and poorly characterized. We describe two cases (55-year-old male with 7.5 cm renal mass and 32-year-old female with 5.5 cm mesenteric mass) illustrating their misleading immunophenotypes. Histologically, both showed eosinophilic and focally clear epithelioid cells arranged into sheets, nests, and trabeculae. Immunohistochemistry showed ALK, EMA, and AE1/AE3 immunoreactivity suggesting *ALK*-rearranged renal cell carcinoma (Case 1) and coexpression of keratin, EMA, synaptophysin, and chromogranin-A, suggesting neuroendocrine neoplasm (Case 2). Targeted RNA sequencing revealed *EWSR1-CREM* (Case 1) and *FUS-CREM* (Case 2) fusions. These cases add to the spectrum of *CREM* fusion-positive intra-abdominal epithelioid neoplasms. Their unusual immunophenotype and unexpected sites represent major pitfalls, underline a wide differential diagnosis, and emphasize the value of molecular testing in correctly diagnosing them.

## Introduction

Gene fusions involving the Ewing Sarcoma Breakpoint Region 1 gene (*EWSR1* on 22q) and one of the CREB family genes (*CREB1*, *ATF1*, and *CREM*) have been increasingly recognized in soft tissue and visceral neoplasms with significant phenotypic and demographic diversity [1]. The major representatives of this category are clear cell sarcoma (CCS) of tendon and aponeuroses (mainly *EWSR1-ATF1* fusions), malignant gastrointestinal neuroectodermal tumor (mainly *EWSR1-CREB1* fusions), angiomatoid fibrous histiocytoma (mainly *EWSR1-CREB1* fusions), and clear cell carcinomas of the head and neck (*EWSR1-ATF1* fusions) [1]. As a group, these neoplasms differ significantly in morphology, immunophenotypes, and behavior, ranging from low-grade indolent (e. g., angiomatoid fibrous histiocytoma) to aggressive (e.g., CCS) [1]. Within the *CREB* fusion family, *CREM-*rearranged neoplasms are rare. They include subsets of CCS, angiomatoid fibrous histiocytomas, and clear cell carcinoma of the head and neck [2]. We describe two *CREM*-rearranged intra-abdominal malignant epithelioid neoplasms (one renal and one mesenteric) displaying confusing immunophenotypes: expression of ALK and keratin (Case 1) and keratin and neuroendocrine markers (Case 2). In both, diagnosis was only made after NGS testing uncovered the underlying fusions.

## Materials and methods

Cases were retrieved from our consultation files. Case 2 was identified among 25 mesenchymal/non-epithelial neoplasms in a cohort of 346 neuroendocrine mimics (Kasajima et al., submitted). Immunohistochemistry (IHC) was performed on 3-µm sections using a fully automated system (“Benchmark XT System,” Ventana Medical Systems Inc.) and the following antibodies: keratin (AE1/AE3, 1:40, Zytomed), vimentin (V9, 1:100, Dako), PAX8 (rabbit polyclonal, 1:50, Cell Marque), CK7 (OV-TL, 1:1000, Biogenex), CK5 (XM26, 1:50, Zytomed), TTF-1 (8G7G3/1, 1:500, Zytomed), ERG (EPR3864, prediluted, Ventana Medical Systems), CD31 (JC70A, 1:20, Dako), CD10 (56C6, 1:20, Dako), p63 (SFI-6, 1:100, DCS), desmin (D33, 1:250, Dako), smooth muscle actin (1A4, 1:200, Dako), S100 protein (polyclonal, 1:2500, Dako), CD34 (BI-3C5, 1:200, Zytomed), CD30 (Ber-H2, 1:40, Zytomed), MUC4 (EP256, 1:500, Epitomics), TLE1 (polyclonal, 1:200, Santa Cruz), STAT6 (sc-621, 1:1000, Santa Cruz), SMARCB1/INI1 (MRQ-27, 1:50, Zytomed), synaptophysin (MQR40, 1:1, Ventana/Roche), chromogranin-A (LK2H10, 1:500, Thermo Fisher), SDHB (polyclonal, 1:200, Sigma-Aldrich), TFE3 (clone MRQ-37, 1:100, Cell Marque), and MUC1 (MRQ17, 1:50, Cell Marque) according to the manufacturer instructions. FFPE tissue from Case 2 was studied by electron microscopy (LEO 912, Zeiss, Oberkochen, Germany) with digital image device (TRS Tröndle, Moorenweis, Germany) according to routine methods.

### Molecular testing

RNA-based NGS was performed as described previously [3]. *ALK/EWSR1* (Case 1) and *FUS/TFE3* (Case 2) FISH analyses were performed using ZytoLight® SPEC Dual Color Break Apart Probes (ZytoVision, Bremerhaven, Germany) designed to detect translocations involving these genes, respectively. Case 2 was tested for *SDHB* mutations using the methods described previously [4].

## Results

### Case histories

#### Case 1

A 55-year-old male underwent radical nephrectomy for a renal mass. His medical history was negative for other malignancies. He is currently diagnosed with metastatic disease in the pelvis 23 months from initial diagnosis.

#### Case 2

A 32-year-old female without other primary malignancy presented with a mesenteric mass that was resected together with the adjacent small bowel segment. She presented 8 months later with peritoneal, pleural, and lymph node metastases. Currently (13 months from initial diagnosis), she is alive with extensive progressive disease under palliative chemotherapy.

### Pathological findings

#### Case 1

**Fig. 1:**
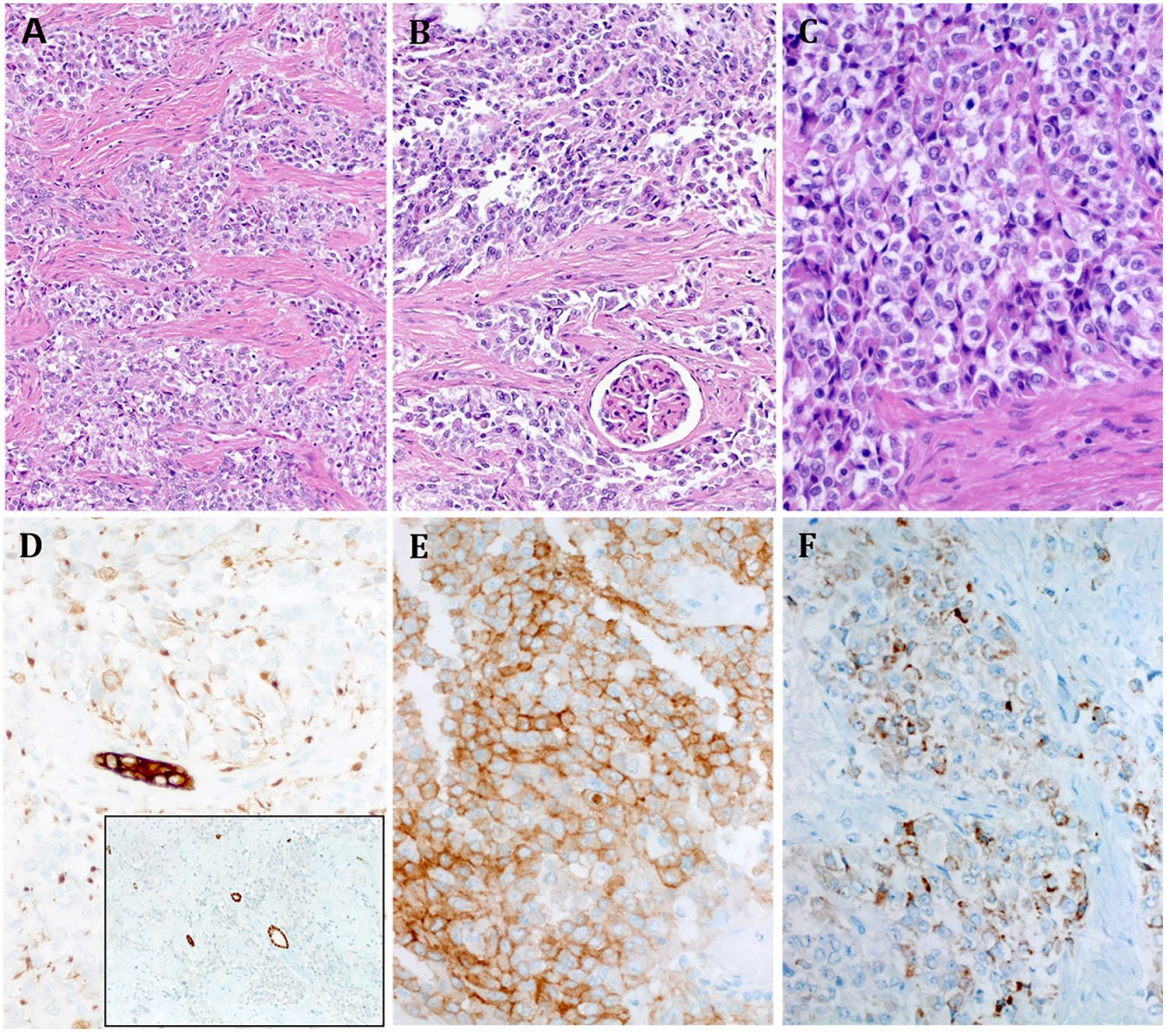
Representative histological images of Case 1 show epithelioid cells disposed into communicating irregular nests within fibrous stroma (**A**) with focal entrapment of the glomeruli (**B**). **C** At high power, the epithelioid morphology is seen; note the variably granular eosinophilic to clear cytoplasm. **D** AE1/AE3 reveals mainly paranuclear dot-like pattern (note the strong expression in entrapped tubules; main image). **D**, inset, PAX8 highlights entrapped tubules, but the tumor cells are negative. **E** Strong cytoplasmic ALK expression is seen. **F** MUC4 is variably positive

The nephrectomy specimen contained a well-demarcated 7.5 cm renal medullary mass, distinct from the renal pelvis without extrarenal extension. Histological examination showed medium-sized epithelioid cells disposed into communicating nests and trabeculae within a prominent fibrous stroma, entrapping glomeruli and tubules (Fig. 1A, B). The nuclei were moderately vesicular with small inconspicuous nucleoli surrounded by granular eosinophilic to clear cytoplasm (Fig. 1C). A few rhabdoid cells were seen. Necrosis was absent. < 4 mitoses/10 HPFs were found. IHC showed diffuse expression of vimentin, EMA, AE1/AE3 (Fig. 1D), cytoplasmic ALK (Fig. 1E), and variably (40% of cells) MUC4 (Fig. 1F). All other markers listed above including PAX8 (Fig. 1D inset) were negative. SMARCB1 expression was retained.

#### Case 2

**Fig. 2:**
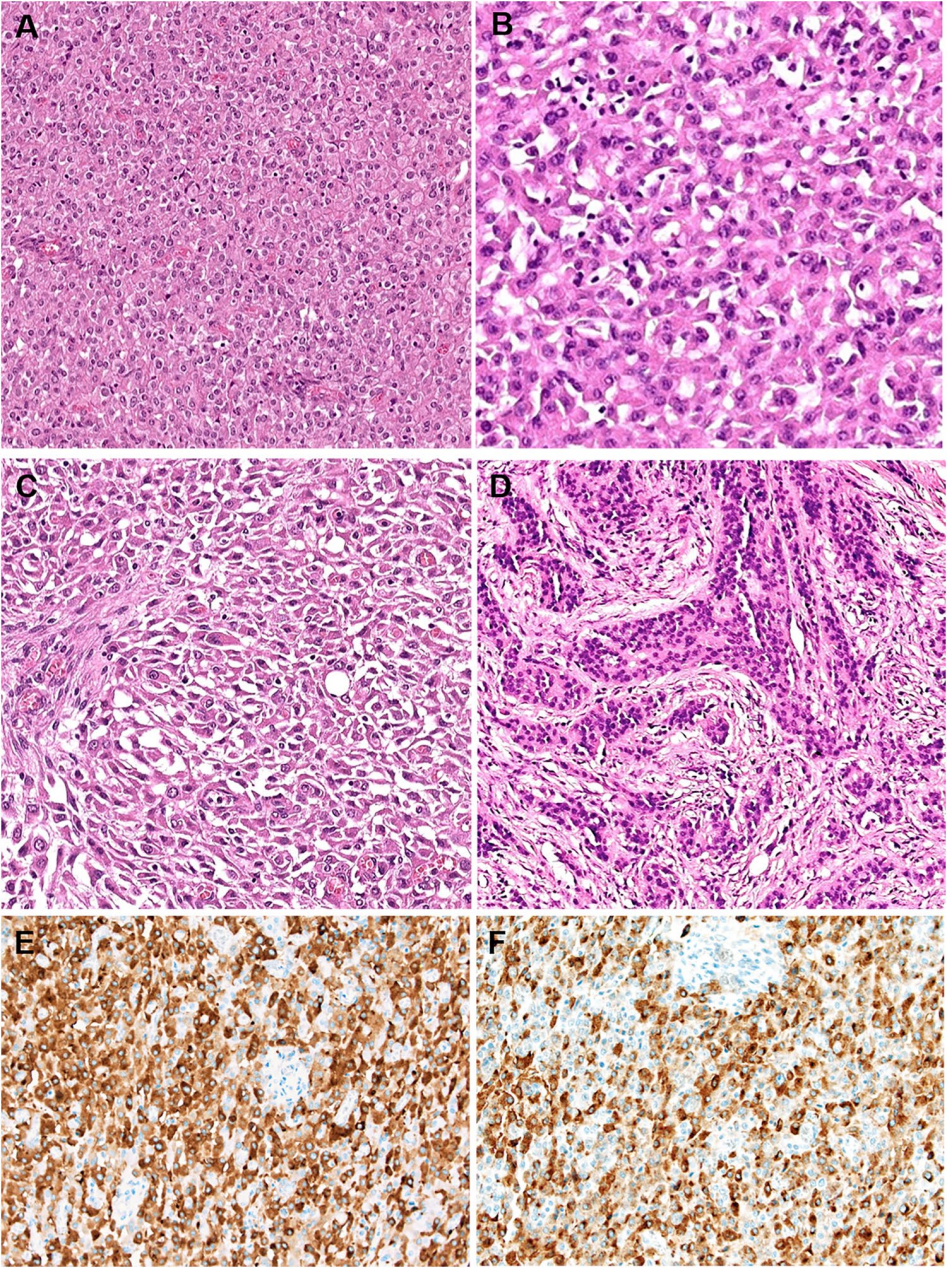
Case 2 showed medium-sized epithelioid cells forming solid sheets lacking a distinct stroma with variable cytoplasmic clearing (**A**, **B**). **C** Focal areas displayed rhabdoid or spindle-shaped morphology. **D** A pseudotrabecular pattern within desmoplastic stroma was seen focally. Immunohistochemistry revealed strong expression of synaptophysin (**E**) and chromogranin-A (**F**)

The tumor measured 5.5 cm and was intra-mesenteric distinct from the gut wall. Histological examination showed medium-sized epithelioid cells forming well vascularized sheets with variably eosinophilic, clear (Fig. 2A, B), rhabdoid, and spindled (Fig. 2C) cell morphology lacking a distinct stroma. Focal pseudotrabecular structures within desmoplastic stroma were noted (Fig. 2D). Fifteen mitoses were observed in 10 HPFs. Hemorrhagic necrosis was present. IHC revealed strong positivity for AE1/AE3, CK18, synaptophysin (Fig. 2E), chromogranin-A (Fig. 2F) and MUC1, and loss of SDHB. TFE3 showed diffuse moderate reactivity. SMARCB1 expression was retained. Repeated electron microscopy revealed no cytoplasmic membrane bound neurosecretory granules.

### Molecular findings

**Fig. 3:**
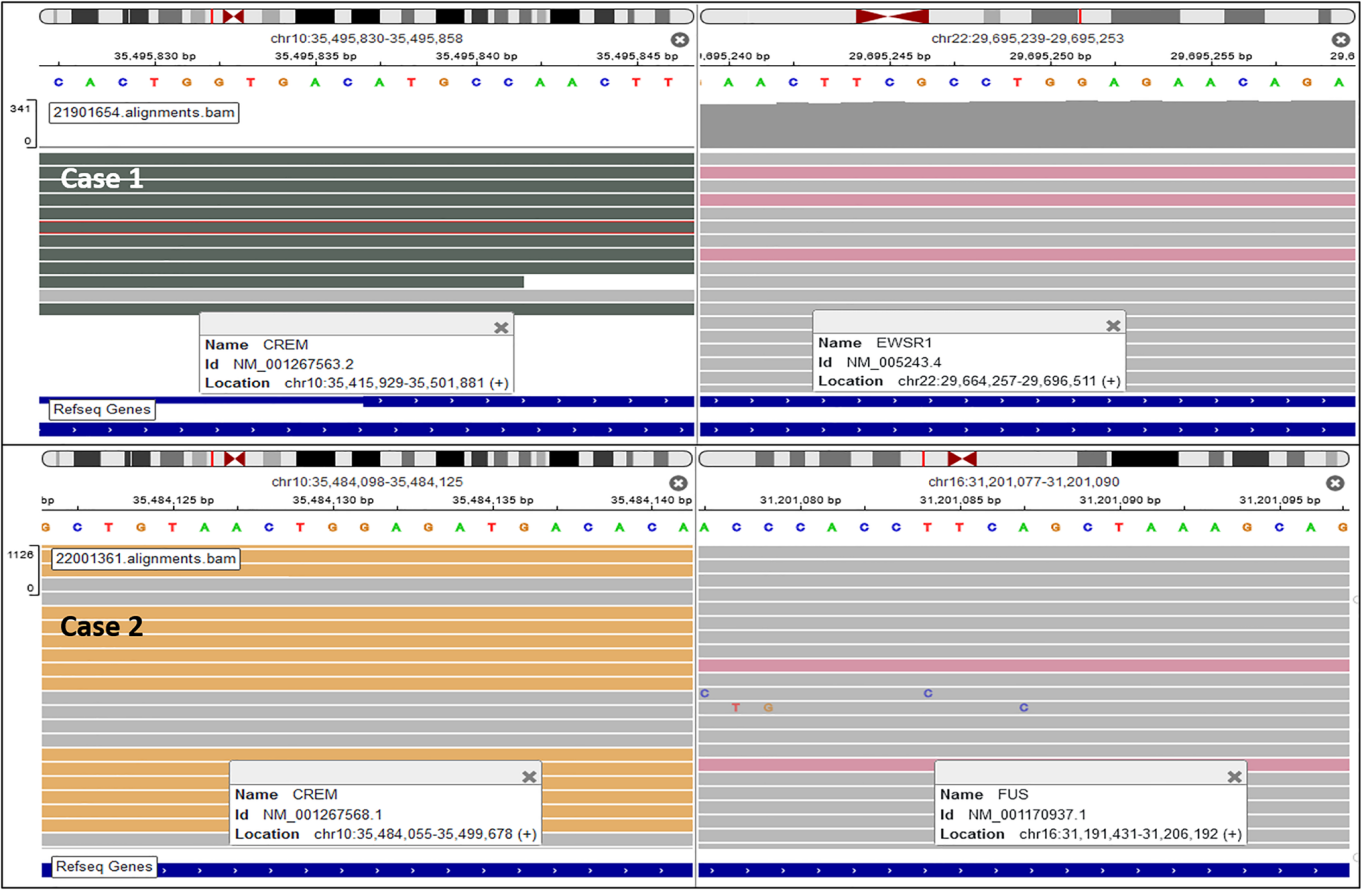
IGV split-screen view of read alignments of the identified *CREM-EWSR1* (Case 1) and *CREM-FUS* (Case 2) fusion events. Shown are the breakpoints in the *CREM* (left) and the *EWSR1/FUS* (right) genes, respectively. Alignments whose mate pairs are mapped to the fusion sequence on the other chromosome are colored in dark green/pink for *CREM-EWSR1* and ocher/pink for *CREM-FUS*. All other alignments are colored gray

With a provisional diagnosis of ALK + renal cell carcinoma (RCC), ALK FISH failed to show rearrangement or copy number gains in Case 1. Targeted RNA sequencing revealed a *EWSR1-CREM* fusion (Fig. 3), which was confirmed by *EWSR1* FISH. Case 2 (mesenteric tumor) showed a *FUS-CREM* fusion (Fig. 3) which was also confirmed by *FUS* FISH. NGS (Case 2) revealed no *SDHB* mutation (which was suggested by the immunohistochemical loss of the protein).

## Discussion

With increasing use of targeted RNA NGS in routine surgical pathology practice, the numbers and variants of gene fusions detected in neoplasms have been increasing, and several new molecularly defined entities are emerging. *EWSR1/FUS-CREM* fusions were recognized recently in a group of unclassified epithelioid mesenchymal neoplasms showing predilection for intra-abdominal organs, not fitting any known *EWSR1*-*CREB*-rearranged entity [1, 2]. We herein expand the topographic and phenotypic features of these emerging neoplasms and highlight increasing pitfalls associated with them. Including our cases, 12 *EWSR1/FUS-CREM*-positive intra-abdominal epithelioid neoplasms have been reported [2, 5, 6]. Affected sites were omentum/mesentery (4), stomach (2), kidney (2), pelvic organs (2), adrenal (1), and unspecified site (1). Seven tumors harbored *EWSR1-CREM* and five *FUS-CREM* fusions [2, 5, 6]. Very few examples occurred at extra-abdominal soft tissue (chest wall) or visceral (lung) sites [2, 7]. These neoplasms possess a definite malignant potential with propensity for peritoneal recurrences and nodal and distant metastasis [2, 5, 6].

The morphology of these tumors seems distinctive. They all displayed monotonous epithelioid morphology with variable cytoplasmic clearing and sparse fibrous stroma. Most cases expressed keratin and EMA [2, 5, 6]. ALK was positive in one (intra-abdominal [2]), synaptophysin in two (chest wall and lung [2, 7]), and MUC4 in two (chest wall and gastric [2, 6]) previous cases. We add the expression of ALK, MUC4, and synaptophysin in one case each and report novel chromogranin-A expression in one.

Although the MUC4-positive gastric tumor was diagnosed as sclerosing epithelioid fibrosarcoma (SEF), the depicted histology suggests similarity to the current cases with variable clear cell epithelioid pattern, lacking characteristic sclerosis of SEF [6]. Another reported *EWSR1-CREM*-positive SEF was not illustrated [8]. Including our cases, 3/5 tumors tested positive for MUC4. This finding is relevant given that MUC4 expression and presence of *EWSR1* fusion by FISH are otherwise considered diagnostic of SEF in the context of epithelioid mesenchymal neoplasms [9]. However, distinction of the two entities seems justified to address their potentially different biology. Most SEFs are driven by *EWSR1-CREB3L1* and rarely by *FUS-CREB3L1* fusions [1].

Our renal case expands the spectrum of *EWSR1*-rearranged renal sarcomas and represents the second *EWSR1-CREM* fusion-positive renal case [5]. Moreover, it represents true pitfall as detection of ALK in eosinophilic RCC strongly suggests an *ALK*-rearranged RCC [10]. Indeed, this was our initial diagnosis. The absence of PAX8 suggests alternative diagnoses and justifies further molecular testing in such a case. ALK expression is common in several translocation sarcomas including angiomatoid fibrous histiocytoma [11] and rhabdomyosarcoma [12]. The molecular basis of ALK immunoreactivity observed in diverse fusion-associated soft tissue tumors seems heterogeneous and associates with specific types of fusions (e.g., *EWSR1/FUS-TFCP2* and *EWSR1* but not *MEIS1-NCOA2* and other fusions [11, 12]). While the ALK protein expression in alveolar rhabdomyosarcoma and neuroblastoma correlates frequently with *ALK* copy number gains, none of angiomatoid fibrous histiocytoma showed this feature [11]. ALK might represent a downstream target of EWSR1 and PAX-FOXO1 fusion proteins, but this remains speculative [11, 12].

Regarding Case 2, it is noteworthy that it labeled not only for synaptophysin but also for chromogranin-A, a combination that we encountered only rarely in mesenchymal neoplasms with neuroendocrine features (data not shown). Since chromogranin-A normally resides in the membrane of neurosecretory granules, we studied the tumor tissue by electron microscopy to search for neurosecretory granules but could not identify any. We conclude that chromogranin-A protein associates with other cell organelles than secretory granules in this neoplasm. Loss of SDHB in this case remained unexplained and represents a further confusing factor with neuroendocrine neoplasms. Finally, aberrant or equivocal TFE3 expression noted in Case 2 and in a previous case [7] may suggest a *TFE3*-rearranged neoplasm. This is particularly important in renal cases as rare Xp11-translocation RCC may harbor *EWSR1-TFE3* fusions and be indistinguishable from *EWSR1/FUS-CREM* neoplasms by FISH alone [13]. All these observations underline the morphological and immunophenotypic heterogeneity of *EWSR1/FUS-CREM* fusion driven mesenchymal neoplasms.

In summary, we report two intra-abdominal malignant epithelioid mesenchymal neoplasms carrying the recently described *EWSR1-CREM* and *FUS-CREM* fusions. Besides their epithelioid morphology, these tumors displayed unusual misleading immunophenotypes. Recognition of these findings should help to avoid misinterpretation as SEF, *ALK*-rearranged RCC, and neuroendocrine carcinoma. The cases highlight the value of precise genotyping in these challenging lesions to ovoid misdiagnoses.
